# A strategy for high-yield lauric acid production in *Hermetia illucens* fed with pre-fermented sweet potato-based substrate

**DOI:** 10.1186/s40643-026-01085-6

**Published:** 2026-06-12

**Authors:** Vivek Manyapu, Yo-Chia Chen

**Affiliations:** 1 Department of Tropical Agriculture and International Cooperation, National Pingtung University of Science and Technology, Pingtung, 912 Taiwan, ROC; 2 Department of Biological Science and Technology, National Pingtung University of Science and Technology, Pingtung, 912 Taiwan, ROC

**Keywords:** Bacterial fermentation, β–oxidation, de novo synthesis, Glycolysis, *Klebsiella*, Precursors, Pre-treatment

## Abstract

The intensification of animal farming is associated with increased infectious and zoonotic disease risks. The black soldier fly larvae (BSFL) have antimicrobial activity that could potentially be used to suppress diseases and improve animal health. This study used carbohydrate-rich sweet potato and lauric acid-rich desiccated coconut to differentiate the primary source of lauric acid. The study also investigated the effect of pre-fermenting the substrates with *Bacillus* sp. and *Klebsiella* sp. on the BSFL growth performance and fatty acid composition, particularly on lauric acid. Correlation analyses were conducted between the larvae fed with fresh substrate and fermented substrate. Overall, the sweet potato enhanced the lauric acid biosynthesis, and the fermented sweet potato-based substrate resulted in a remarkable lauric acid production of 82%, i.e., 365 mg g^−1^ in the BSFL biomass. Pre-fermentation aided production of precursors like oleic acid, linoleic acid, and glycolysis of readily available carbohydrates, which spiked the lauric acid biosynthesis.

**Supplementary Information:**

The online version contains supplementary material available at 10.1186/s40643-026-01085-6.

## Introduction

The sustainable approach for the valorization of bioresources is the key to the current era. The high-value outputs or byproducts are mandated to be the result of bioprocess engineering and/or circular bioeconomy. One such trending bioactive compound is lauric acid (LA), a 12-carbon medium–chain saturated fatty acid, chemically named as dodecanoic acid (C_12_H_24_O_2_). There are wide agricultural and industrial applications of LA, such as soap and shampoo production (sodium lauryl sulphate), biodiesel production (LA as precursor), pharmaceutical industries (antimicrobial, anti-cancer), in textile industries (LA as lubricant), food production (LA as emulsifier), animal feed production (for pisciculture, poultry and other livestock) etc. (Suryati et al. 2023).

The natural sources of LA are derived from plant oils (palm kernel oil, coconut oil, ucuuba butter, babasu oil, many more) (Suryati et al. 2023). However, the agronomical practices impose a huge challenge. Although there are some animal fats that source LA in lower quantities, the oil from black soldier fly larvae (BSFL) is gaining attention. With a suitable substrate, for example, a carbohydrate-rich diet or fruit-based feed, the BSFL can produce LA content that can be up to 73.6% (Manyapu and Chen 2026) and 76.4% (Leong et al. 2015). Though this larva has wide applications such as biofuel production, composting, etc., its use as protein feed for farm and pet animals has a greater influence (Siddiqui et al. 2024).

As the demand for animal-based protein (chicken, pork, fish, meat, milk) is increasing rapidly, animal farming has become more intensified, and the population density of animals has led to a situation that is conducive to the spread of infectious agents (Stevenson 2023). The increasing incidence of diseases has now become a major threat to animal health and welfare, and it also has the potential for zoonotic transfer, which affects food security and the health of humans (Juhas 2023). The BSFL with a high-grade antimicrobial property has the potential for the suppression of zoonotic pathogens.

Recent studies on animal nutrition showed improved nutrient balance, gut health, and immune resilience through antioxidant and metabolic stability with partial substitution of animal diets with BSFL meal or BSFL oil (de Souza Vilela et al. 2021; Ma et al. 2025; Noh et al. 2026; Yang et al. 2026). In addition to immune-modulation, BSFL also has the potential to alter the methanogens’ abundance in the ruminants, leading to reduced emission of CH_4_ (Jayanegara et al. 2021). Adding BSFL oil rich in lauric acid at a rate of 4% into the cattle diet reduced methane emission without affecting digestibility, whereas 6% BSFL oil reduced dry matter degradability and affected rumen microflora in vitro (Prachumchai and Cherdthong 2023).

However, the production of BSFL with higher levels of LA is challenging, especially due to the dependency on the substrates provided to the larvae since nutrient availability affects the lipid synthesis and growth performance (Gatlin et al. 2024). Optimizing BSFL production for high LA content requires careful management of diet formulation and extraction processes while balancing larval growth and nutritional content trade-offs. Additionally, microbial fermentation supports the conversion of carbohydrates into organic acids or alcohols, and protein molecules into amino acids or peptides (Memon et al. 2025a) that can act as precursors for the BSFL LA biosynthesis. Therefore, the present study links this critical gap by leveraging carbohydrate-driven de novo synthesis and bacterial pre-fermentation to achieve a remarkable lauric acid enrichment. A novel *Bacillus-Klebsiella* consortium was applied for a strategic bioconversion of complex substrates into intermediate precursors, unravelling a controlled and reproducible pathway for the biosynthesis of high-grade LA. Moreover, this study introduces the Carbohydrate-to-lauric acid conversion ratio (CLR) as an innovative quantitative index to assess and enhance substrate efficiency in BSFL-based fatty acid production. Eventually, these findings would provide insights for establishing a scalable and eco-friendly biorefinery strategy for the production of antimicrobial-enriched BSFL biomass with high pertinency in livestock production, pharmaceutical, and bioproduct industries.

## Materials and methods

### Isolation, screening, and identification of bacteria

Using a sterile scalpel, a fresh sweet potato (obtained from Neipu fresh market, Taiwan) and corn silage were cut into small pieces and soaked in 2.5% brine solution (Stoll et al. 2020) for 3 days in separate air-tight glass bottles. Using the serial dilution method, the bacteria were isolated over De Man–Rogosa–Sharpe (MRS) agar. The pure cultures were transferred into LB agar. The single colonies from the LB agar were tested for lipase (phenol red agar) (Singh et al. 2006), cellulase (carboxymethyl cellulose), xylanase (birchwood xylan) (Meddeb-Mouelhi et al. 2014), protease (skim milk agar) (Mushtaq et al. 2023), amylase (Sharif et al. 2023), and pectinase (pectin agar) (Kabir and Tasmim 2019). The three potential bacteria with appreciable enzyme activity were screened out, and through 16S rRNA Sanger sequencing, the bacteria were identified. The identification was supported by morphological parameters like Gram-staining, hanging drop motility test, capsule test (Anthony’s capsule stain), lactose fermentation (eosin methylene blue agar), hemolysis test (blood agar), catalase test (hydrogen peroxide), and Kovac’s Indole test.

## Substrate preparation, BSFL rearing, and experimental design

Fresh sweet potato, minced pork meat, and minced pork fat were acquired from a local market in Neipu township, Taiwan. Desiccated coconut was procured from Wang-Lai wholesale shop, Neipu, Taiwan. The corn flour and wheat bran were obtained from Dali feed factory, Pingtung, Taiwan. The black soldier fly eggs were received from HQ Biotech Co., Ltd., Taichung, Taiwan. The eggs after hatching were reared on overnight-soaked wheat bran. The 6-day-old larvae were used for the BSFL treatment. The bioconversion experiment was carried out in the net house inside the campus of the National Pingtung University of Science and Technology, Taiwan (22.648755 N, 120.605942 E). Two experimental sets were prepared: fresh substrates (Set-1) vs. pre-fermented substrates (Set-2). The six fresh substrate treatments, namely C, T1, T2, T3, T4, and T5, denoted as Set-1, were prepared according to the composition indicated in Table 1. Another batch of Set-1 was fermented with bacterial consortium in zip-lock bags (Set-2), named as PC, PT1, PT2, PT3, PT4, and PT5, incubated at 37 °C for 3-days with occasional mixing. The bacterial consortium was prepared by mixing three bacterial cultures grown in Luria-Bertani broth for 24 h at 37 °C. The three cultures, namely CS231, LSP487, and SSP487, were used individually at around 1.67 × 10^6^ CFU g^−1^ of wet substrate, for a total of around 5 × 10^6^ CFU g^−1^ of wet substrate. All twelve treatments from the two sets (Set-1 and Set-2) were prepared in triplicate with an individual wet weight of 317 ± 4 g, and an average MC of 65%. The feed replicates from the two sets were loaded in 1 L PET berry packaging boxes fitted with fruit mesh on the top to inhibit larval escape. The 6-day-old BSFL were added to each treatment at the rate of 100 per box, corresponding to 1.33 larvae cm^−2^. The BSFL bioconversion was carried out up to 12 days, when the first pre-pupae appeared.

**Table 1:** Experimental design and the distribution of different substrates among the treatments

Treatments	C	T1	T2	T3	T4	T5
Corn flour	30%					
Sweet potato		0%	20%	30%	40%	60%
Desiccated coconut		60%	40%	30%	20%	0%
Pork meat		10%	10%	10%	10%	10%
Pork fat		10%	10%	10%	10%	10%
Wheat bran	70%	20%	20%	20%	20%	20%

## Physico-chemical analysis of substrate and BSFL

The pH was measured at every 3-day interval for up to Day-12. The initial fresh substrates, the pre-fermented substrates, the final residual substrates after BSFL treatment, the 6-day old larvae and the matured larvae after 12 days of treatment were partially defatted for calculating crude protein (CP) taking Kp value 5.6 for substrates (Mariotti et al. 2008) and 4.76 for larvae (Janssen et al. 2017), ash content, total organic carbon (TOC) (Nelson and Sommers 1996). The moisture content (MC), crude fat (CF), nitrogen-free extract (NFE), and gross energy content (GEC) were estimated according to Manyapu and Chen (2026). Chitin for the BSFL was estimated using the formulae N% × (6.25–4.76) (Young Kim et al. 2024). The fatty acid composition of the raw materials was estimated before the commencement of the experiment (Table S1).

## BSFL growth performance and bioconversion indices

The survival rate (SR) %, substrate reduction index (SRI), specific growth rate (SGR), protein conversion efficiency (PCE), and bioconversion efficiency (BCE) were estimated according to Zhang et al. (2025) with some modifications to PCE and BCE. Overall digestion (OVD) was calculated according to Yeow et al. (2024). The carbohydrate-to-lauric acid conversion ratio (CLR) has been evaluated in this study to relate the BSFL LA production to the substrate carbohydrate content.

1 $$ {\mathrm{SR}}\left( \% \right){\text{ = }}\frac{{{\mathrm{LNf}}}}{{{\mathrm{LNi}}}} \times {\mathrm{100}} $$

2 $$ {\mathrm{SRI}}\left( \% \right){\text{ = }}\frac{{{\mathrm{SWi}} - {\mathrm{SWf}}}}{{{\mathrm{SWi}} \times {\mathrm{T}}}} \times {\mathrm{100}} $$

3 $$ {\mathrm{SGR}}\left( \% \right){\text{ = }}\frac{{{\mathrm{ln}}({\mathrm{LWf}}) - {\mathrm{ln}}({\mathrm{LWi}})\:}}{{{\mathrm{Number}}\:{\mathrm{of}}\:{\mathrm{days}}\:{\mathrm{of}}\:{\mathrm{treatment}}}} \times {\mathrm{100}} $$

4 $$ {\mathrm{PCE}}\left( \% \right){\text{ = }}\frac{{{\mathrm{LWf}} \times {\mathrm{LCP}}\% \times {\mathrm{SR}}\% }}{{{\mathrm{SWf}} \times {\mathrm{SCP}}\% }} \times {\mathrm{100}} $$

5 $$ {\mathrm{BCE}}\left( \% \right){\text{ = }}\frac{{({\mathrm{LWf}} - {\mathrm{LWi}}) \times {\mathrm{SR}}\% }}{{{\mathrm{SWi}} - {\mathrm{SWf}}}} \times {\mathrm{100}} $$

6 $$ {\mathrm{OVD}}\left( \% \right){\text{ = }}\frac{{{\mathrm{SWi}} - {\mathrm{SWf}}}}{{{\mathrm{SWi}}}} \times {\mathrm{100}} $$

7 $$ {\text{CLR = }}\frac{{({\mathrm{LWf}} \times {\mathrm{SR}}\% \times {\mathrm{LAf}}) - ({\mathrm{LWi}} \times {\mathrm{100}} \times {\mathrm{LAi}})}}{{({\mathrm{SCi}} \times {\mathrm{SWi}}) - ({\mathrm{SCf}} \times {\mathrm{SWf}})}} $$

where LNf and LNi represent the final and initial number of larvae, SWf and SWi represent the final and initial substrate DM (g), T represents the duration of bioconversion in days, LWf represents the final larval DM (g), LWi represents the initial 6-day-old larval DM (g), LCP% and SCP% represent crude protein % of the final larvae, and the final substrate, LAf and LAi represent lauric acid content (mg g^−1^) in the final and initial larvae, SCi and SCf represent the initial and final substrate carbohydrate content.

## Fatty acid analysis estimation using GC-MS

From the method transcribed by Hara and Radin (1978), the lipids were extracted, methylated into fatty acid methyl esters (FAMEs), and filtered using a syringe filter (0.20 μm). A Shimadzu GC–MS QP2010 equipped with a DB-5HT capillary column (30 m × 0.25 mm i.d., 0.1 μm film thickness; Agilent Technologies, USA) was used for the FAME analysis. The carrier gas was Helium flowing at 1 mL min^−1^. 1 µL sample was injected in split mode (20:1) at 250 °C. The oven temperature was initiated with 50 °C (held for 1 min), ramped to 200 °C at 10 °C min^−1^ (no hold), then to 280 °C at 3 °C min^−1^ with a final hold of 15 min. The resultant fatty acids were identified and compared with the retention times obtained from the standard mixture Supelco 37 FAME Mix (Merck, Poole, UK). The individual fatty acids were expressed as mg g^−1^ dry weight of the material.

### Statistical analysis

All the data (*n* = 3) were processed with IBM SPSS software version 20.0 (IBM Corp., Armonk, NY, USA) for two-way ANOVA and Tukey HSD tests (*p* < 0.05). An independent t-test was analysed using MS-Excel at *p* < 0.005, *p* < 0.01, *p* < 0.05 for comparing specific fatty acid content in fresh vs. fermented substrate. The bi-directional bar charts were prepared using Kutools for Excel version 34.00 in MS-Excel. The bar graphs and principal component analysis (PCA) were generated with GraphPad Prism (version 11.0.0 (84), LLC, Boston, MA, USA). Pearson’s correlation coefficients were analysed for correlation analysis. The double matrices correlation map and heat map with dendrogram were constructed using Chiplot (https://www.chiplot.online/). The phylogenetic tree was constructed using MEGA version 12.1.2.

## Results

### Screening and identification of bacteria for an effective consortium

A total of 11 bacterial isolates were initially obtained from two sources (fermented sweet potato and fermented corn silage) and assessed for suitability as pre-fermentation screening agents based on their functional hydrolytic activity, substrate specificity, and complementary metabolic potential (Table S2). The selection was based on bacterial isolates showing (i) the production of multiple enzymes relevant to the experimental substrate (carbohydrate, protein, and lipid fractions), (ii) high enzyme index values, and (iii) stable and consistent bacterial growth patterns. Three bacterial isolates showing the most complementary enzyme profiles were selected and purified by repeated streaking. The CS231 isolate, with Gram-positive, highly motile rod-shaped cells, formed dry, irregular colonies (Fig. S1). The isolate was characterized by high amylase, protease, pectinase, and cellulase activity, negative lactose fermentation, and α–hemolysis effect (partial hemolysis) on blood agar (Fig. S1, Table S3), a characteristic consistent with non-pathogenic members of the *Bacillus* group. On the other hand, the LSP487 and SSP487 isolates with Gram-negative, rod-shaped cells formed smooth, slightly mucoid colonies (Fig. S1). Both the LSP487 and SSP487 isolates showed high lipase, cellulase, and moderate pectinase activity, and a high lactose fermentation capacity, identifying them as lactose-fermenting members of the Enterobacteriaceae family (Table S2). Therefore, the CS231 was chosen for its potential to break down complex carbohydrates and proteins, while LSP487 and SSP487 were selected for fermentation properties (to utilize the simpler carbohydrates) and also for lipid biodegradation. Thus, the bacterial consortium was hypothesized to convert the complex ingredients from the provided substrates into readily available components for the consumption of BSFL and bring essential changes in fatty acid composition, especially LA.

Based on the phylogenetic analysis (Fig. S2), CS231 clusters within the genus *Bacillus*, while LSP487 and SSP487 are placed within the genus *Klebsiella* (accession numbers PZ017174, PZ017175, and PZ017176, respectively). However, due to the conserved nature of the 16S rRNA gene, species-level identification cannot be confirmed with certainty, and further characterization using whole-genome sequencing or multi-locus sequence typing (MLST) is recommended. Although LSP487 and SSP487 are phylogenetically related to the genus *Klebsiella*, their safety for use in feed applications requires further evaluation. Biosafety assessments, including antibiotic resistance profiling and virulence gene screening (e.g., for wzi, mrkD, uge, ureA), are recommended before any consideration of scaled application. The fermentation conditions (closed system, low pH, competitive exclusion) may limit pathogenic proliferation, but this assumption should be experimentally verified. Future studies should confirm the absence of virulence factors and assess the microbiological quality of the final larval biomass intended for animal or human consumption.

The complementary metabolic profiles of the selected consortium—particularly the amylolytic and proteolytic capacity of CS231 and the lipolytic activity of LSP487/SSP487—were hypothesized to drive the changes in substrate nutritional composition and fatty acid profile described in the following section.

### Effect of fermentation on the proximate parameters and the substrate fatty acids

**Fig. 1:**
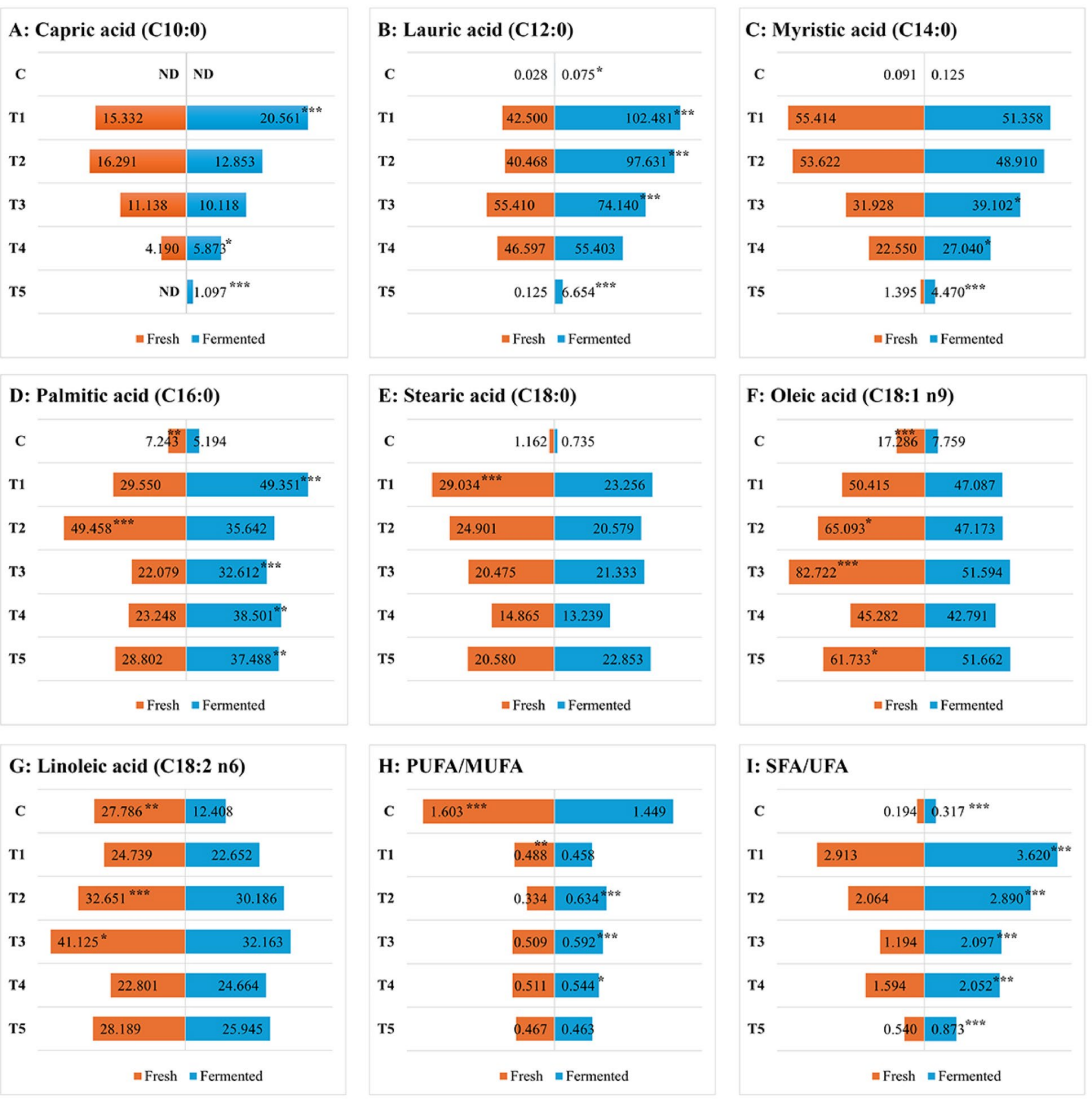
Fatty acids comparison for substrates before and after fermentation. (**A** Capric acid (C10:0), **B** Lauric acid (C12:0), **C** Myristic acid (C14:0), **D** Palmitic acid (C16:0), **E** Stearic acid (C18:0), **F** Oleic acid (C18:1 n9), **G** Linoleic acid (C18:2 n6), **H** PUFA/MUFA, and **I** SFA/UFA); *n* = 3; *** *p* < 0.005; ** *p* < 0.01; * *p* < 0.05; PUFA, polyunsaturated fatty acids; MUFA, monounsaturated fatty acids; SFA, saturated fatty acids; UFA, unsaturated fatty acids

The absence of substantial changes in the MC, CP, CF, and C/N ratios (*p* < 0.05) (Table 2, Table S4) may be explained by the closed fermentation system, which restricts the availability of oxygen and gas exchange. The bacterial fermentation process resulted in a stable proximate composition but selectively increased the usage of unsaturated fatty acids (UFAs), especially oleic acid (C18:1 n9), and linoleic acid (C18:2 n6). As a result, a significantly higher proportion of saturated fatty acids (SFAs), especially from C10:0 to C16:0 was found in pre-fermented substrates compared to the control (Fig. 1). This indicates that the SFAs might have undergone lipolysis and β–oxidation to produce short/medium chain fatty acids (MCFAs). Moreover, the polyunsaturated fatty acids: monounsaturated fatty acids **(**PUFA: MUFA**)** ratio supports SFA synthesis *via* β–oxidation, which could result in acetyl-CoA or malonyl-CoA. The occurrence of SFAs is more visible in fat-rich substrates (T1, T2) after the fermentation. This suggests that the role of carbohydrates is more pronounced and might be the key to LA biosynthesis instead of accumulation from the substrate.

**Table 2:** Substrate parameters after 3 days of fermentation

Treatments	Moisture content (%)	C/*N* ratio^ns^	Crude protein^ns^ (%)	Crude fat (%)	Ash (%)	NFE (%)	GEC (MJ per kg)
C	72.0 ± 2.0^ab^	26.7 ± 6.2	11.5 ± 2.6	5.4 ± 0.5^f^	3.0 ± 0.2^a^	80.2 ± 3.2^a^	18.6 ± 0.2^g^
T1	65.3 ± 3.5^bcd^	30.6 ± 3.7	10.5 ± 1.3	31.6 ± 1.5^bc^	2.1 ± 0.1^bcde^	55.9 ± 2.8^def^	24.6 ± 0.4^bc^
T2	66.7 ± 2.3^bcd^	28.6 ± 7.6	10.1 ± 3.0	40.7 ± 3.7^a^	1.8 ± 0.1^def^	47.4 ± 6.6^f^	26.6 ± 1.0^a^
T3	65.0 ± 2.7^bcd^	30.3 ± 5.0	12.0 ± 2.5	28.7 ± 2.1^c^	2.2 ± 0.2^bc^	57.1 ± 4.9^def^	24.0 ± 0.6^cd^
T4	64.3 ± 2.1^cd^	31.4 ± 6.5	11.2 ± 2.3	18.4 ± 0.4^de^	2.3 ± 0.2^b^	68.1 ± 1.9^bc^	21.6 ± 0.1^ef^
T5	76.0 ± 2.7^a^	33.1 ± 5.9	11.8 ± 1.7	14.6 ± 1.1^e^	1.7 ± 0.1^def^	72.9 ± 2.6^ab^	20.9 ± 0.4^f^
PC	71.3 ± 3.1^abc^	19.4 ± 5.3	16.1 ± 4.7	2.8 ± 0.2^f^	2.1 ± 0.1^bcd^	79.1 ± 4.5^a^	18.5 ± 0.3^g^
PT1	64.7 ± 3.1^bcd^	21.2 ± 4.9	15.4 ± 3.6	34.8 ± 1.4^b^	1.6 ± 0.2^f^	48.1 ± 4.4^ef^	25.7 ± 0.5^ab^
PT2	63.3 ± 3.2^c^	23.6 ± 0.9	14.4 ± 0.7	31.0 ± 2.7^bc^	1.8 ± 0.2^cdef^	52.8 ± 2.6^def^	24.7 ± 0.6^bc^
PT3	62.3 ± 2.5^c^	23.5 ± 4.2	12.6 ± 2.2	27.8 ± 1.4^c^	1.5 ± 0.2^f^	58.2 ± 3.8^cde^	23.9 ± 0.4^cd^
PT4	62.7 ± 0.8^c^	24.8 ± 2.1	14.1 ± 0.9	21.9 ± 1.5^d^	1.7 ± 0.1^ef^	62.3 ± 0.5^bcd^	22.7 ± 0.3^de^
PT5	76.2 ± 1.3^a^	26.1 ± 3.1	13.9 ± 1.1	15.8 ± 0.7^e^	2.2 ± 0.1^bc^	68.1 ± 0.5^bc^	21.3 ± 0.1^f^

The values are expressed in mean ± SD; *p* < 0.05; ns: not significant (*p* > 0.05); NFE: nitrogen-free extract; GEC: gross energy content

Overall, the 3-day bacterial fermentation process favors the formation of short- to medium-chain SFAs (C10–C16), resulting in an increased SFA/UFA ratio in the final product among all treatment combinations. This suggests an application potential of this process in enhancing substances with these particular SFAs, which may be favorable or unfavorable depending on the desired product properties. The lack of an equivalent increase in UFAs suggests that these fatty acids are not major metabolites or do not accumulate in this process.

The selective enrichment of short- to medium-chain SFAs (C10–C16) in pre-fermented substrates, particularly in carbohydrate-rich treatments, was expected to influence both the growth performance and the fatty acid accumulation of BSFL.

### Growth performance and nutritional composition of BSFL

**Fig. 2:**
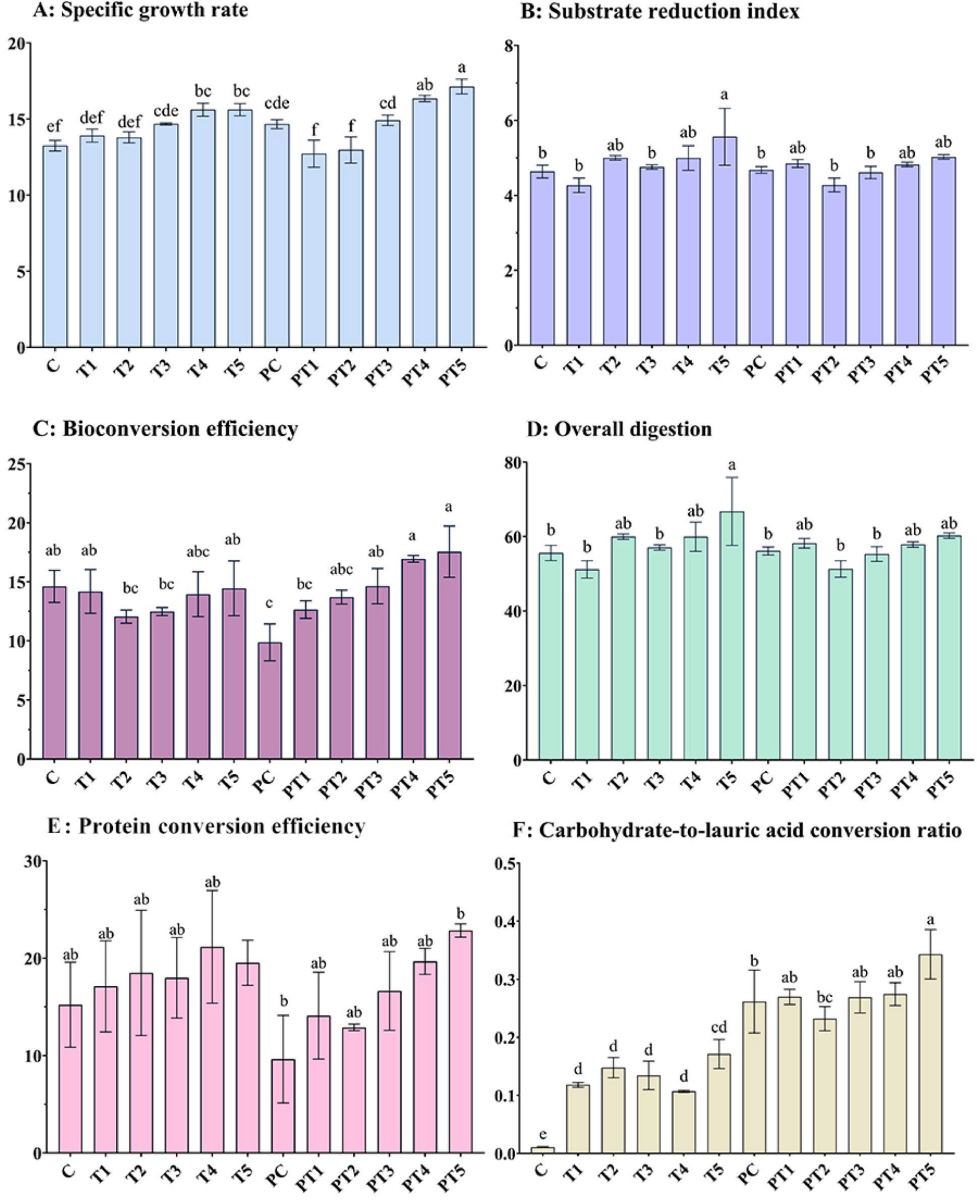
Larval growth and developmental indices. (**A** Specific growth rate; **B** Substrate reduction index; **C** Bioconversion efficiency; **D** Overall digestion; **E** Protein conversion efficiency; and **F** Carbohydrate-to-lauric acid conversion ratio). *n* = 3; *p* < 0.05

The fresh biomass of BSFL ranged from around 156 to 264 mg per larvae (Table 3). The highest biomass increase (around 1980% DM) and survivability (around 89%) were noted in larvae fed with pre-fermented carbohydrate-rich substrates, i.e., high sweet potato content. Among the treatments, the substrates with high carbohydrate content significantly promoted weight gain, survivability, and specific growth rate compared to fat-rich substrates (Fig. 2A).

**Table 3:** The proximate parameters of the mature BSFL after 12 days of treatment

Treatments	Crude protein (%)	Crude fat (%)	Ash (%)	NFE (%)	GEC (MJ per kg)	Chitin (%)	Larvae biomass (mg) (wet weight)	Survival rate (%)
IL^#^	18.8 ± 1.2	4.8 ± 0.4	8.3 ± 0.2	68.1 ± 1.4	18.0 ± 0.2	5.9 ± 0.4	40 ± 2	100 ± 0
C	29.7 ± 1.6^f^	4.7 ± 2.8^h^	5.3 ± 0.1^ab^	60.3 ± 4.2^a^	19.2 ± 0.7^i^	9.3 ± 0.5^f^	175 ± 8^fg^	67 ± 6^bc^
T1	31.9 ± 0.9^ef^	21.8 ± 2.1^g^	4.4 ± 0.2^bcd^	41.9 ± 1.8^b^	23.4 ± 0.4^h^	10.0 ± 0.3^ef^	188 ± 1^def^	72 ± 11^abc^
T2	37.7 ± 1.1^cd^	24.8 ± 2.2^fg^	4.5 ± 0.1^abcd^	33.0 ± 1.5^c^	24.4 ± 0.5^g^	11.8 ± 0.3^cd^	187 ± 2^ef^	78 ± 2^ab^
T3	41.7 ± 1.2^abc^	25.2 ± 1.5^fg^	4.8 ± 0.1^abc^	28.4 ± 1.0^cd^	24.7 ± 0.3^fg^	13.1 ± 0.4^abc^	196 ± 2^cdef^	71 ± 2^abc^
T4	42.1 ± 1.8^abc^	27.7 ± 0.9^ef^	2.6 ± 0.1^e^	27.6 ± 2.1^cd^	25.7 ± 0.3^ef^	13.2 ± 0.6^abc^	224 ± 1^bc^	74 ± 6^abc^
T5	43.1 ± 1.4^ab^	31.1 ± 0.4^de^	4.7 ± 0.6^abcd^	21.2 ± 0.4^ef^	26.1 ± 0.1^de^	13.5 ± 0.4^ab^	237 ± 9^ab^	72 ± 11^abc^
PC	38.9 ± 2.7^bcd^	28.2 ± 0.4^ef^	5.4 ± 0.5^a^	27.5 ± 2.6^cde^	25.1 ± 0.4^fg^	12.2 ± 0.9^bcd^	215 ± 12^bcd^	59 ± 8^c^
PT1	39.9 ± 0.9^bcd^	33.0 ± 0.6^cd^	4.3 ± 0.5^cd^	22.7 ± 2.0^def^	26.4 ± 0.1^de^	12.5 ± 0.3^bcd^	156 ± 12^g^	76 ± 4^abc^
PT2	36.9 ± 2.1^d^	35.3 ± 0.2^c^	4.2 ± 0.5^cd^	23.7 ± 2.2^def^	26.7 ± 0.2^cd^	11.5 ± 0.7^d^	178 ± 17^fg^	84 ± 8^ab^
PT3	36.6 ± 2.6^de^	39.5 ± 1.2^b^	3.8 ± 0.2^d^	20.1 ± 3.1^f^	27.7 ± 0.3^bc^	11.5 ± 0.8^de^	212 ± 6^bcde^	84 ± 2^ab^
PT4	43.5 ± 1.0^ab^	41.1 ± 0.1^ab^	3.8 ± 0.1^d^	11.6 ± 1.2^g^	28.5 ± 0.1^ab^	13.6 ± 0.3^ab^	254 ± 8^a^	83 ± 3^ab^
PT5	45.7 ± 0.3^a^	43.8 ± 0.7^a^	4.3 ± 0.2^cd^	6.1 ± 1.0^g^	29.2 ± 0.2^a^	14.3 ± 0.1^a^	264 ± 16^a^	89 ± 4^a^

The values are expressed in mean ± SD; *p* < 0.05; NFE: nitrogen-free extract; GEC: gross energy content; ^**#**^*IL: initial 6-day-old larvae used for the treatment*

In addition, the larvae associated with carbohydrate-rich substrates had significantly higher CP content (*p* < 0.05) compared to those associated with fat-rich substrates, indicating an improvement in protein conversion efficiency. There was a marked increase in CF content from around 5% in initial larvae reaching up to 31% in fresh substrate-fed larvae, and a further increase (44%) in pre-fermented substrate-fed larvae. The highest fat content was noted in larvae fed with pre-fermented carbohydrate-rich substrates, indicating an improvement in lipid biosynthesis. It is noteworthy that pre-fermentation of fat-rich substrates was found to increase fat content in larvae, suggesting an improvement in substrate digestibility and lipid assimilation efficiency.

Ash content was reduced from 8.3% in early-stage larvae to less than 5.5% in mature larvae. Nitrogen-free extract (NFE) was reduced with an increase in substrate carbohydrate content, with the lowest values noted in larvae fed with pre-fermented carbohydrate, in contrast to the control group C_Larvae_, with the lowest GEC value of 19.2 MJ kg^−1^. The GEC was increased with a reduction in NFE, with the highest values noted in larvae fed pre-fermented carbohydrate-rich substrates.

In brief, carbohydrate-rich substrates, especially pre-fermented substrates, promoted larval growth, nutrient assimilation, and lipid biosynthesis, suggesting an improvement in substrate utilization efficiency. The enhanced larval growth and fat content observed with pre-fermented substrates raised a further question of whether these improvements were accompanied by more efficient substrate bioconversion or not.

### Substrate utilization by BSFL

Limited variations were observed in substrate reduction index (SRI) among the treatments within the 12 days (Fig. 2B), but the BCE varied between 9.8 and 17.5 (Fig. 2C). The larvae belonging to the pre-fermented carbohydrate-rich feed achieved the highest efficiency, implying that the availability of simple sugars after pre-treatment enhances the BSFL bioconversion. The OVD was distributed from 51% to 66% and the highest OVD was found from larvae fed with carbohydrate-rich food without fermentation (Fig. 2D). The PCE had an increased flow with the increase in carbohydrate content and fermentation, enhancing the efficiency further up to 23% (Fig. 2E). The carbohydrate conversion into LA was found to be significant in the BSFL from pre-fermented treatments. The highest CLR of 0.35 was found in the case of PT5_Larvae_ (Fig. 2F), with the highest carbohydrate source subjected to fermentation. There was a significant difference in the fresh substrate-treated larvae with the respective pre-fermented group’s larvae, indicating the importance of fermentation for essential conversions and biosyntheses.

The elevated CLR in pre-fermented treatments suggests that de novo fatty acid synthesis, rather than direct substrate accumulation, drives LA enrichment – a hypothesis further explored through the detailed fatty acid profiling and correlation analysis.

### Fatty acid composition and retentions in BSFL biomass

**Fig. 3:**
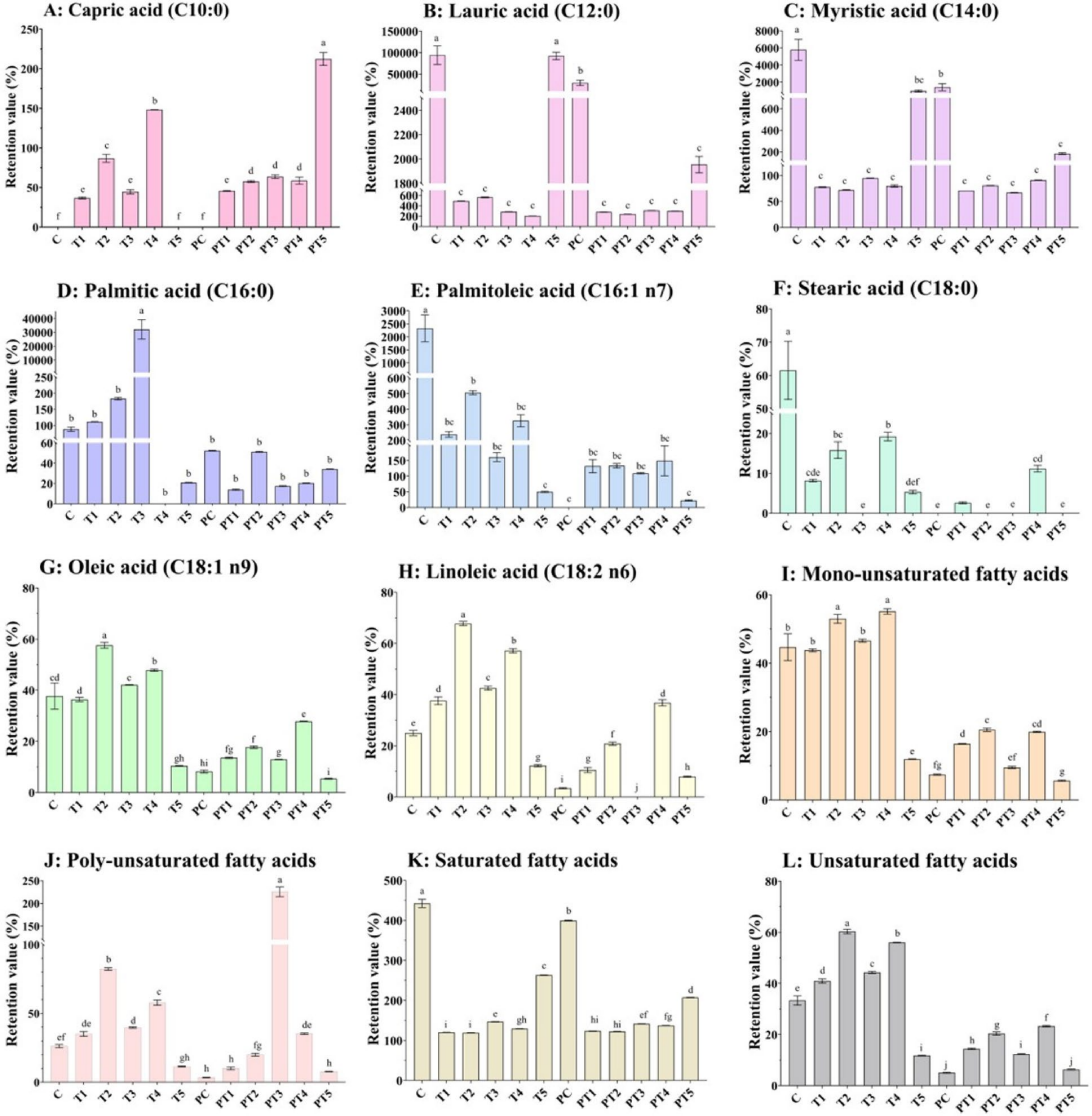
Retention values (%) of fatty acids in the BSFL. (**A** Capric acid (C10:0), **B** Lauric acid (C12:0), **C** Myristic acid (C14:0), **D** Palmitic acid (C16:0), E Palmitoleic acid (C16:1 n7), **F** Stearic acid (C18:0), **G** Oleic acid (C18:1 n9), **H** Linoleic acid (C18:2 n6), and **I** Mono-unsaturated fatty acids, **J** Poly-unsaturated fatty acids, **K** Saturated fatty acids, and **L** Unsaturated fatty acids); *n* = 3; *p* < 0.05

The fatty acid composition of BSFL was significantly influenced by substrate composition and fermentation treatment (Table 4). Saturated fatty acids were found to be dominant in overall fatty acids, with LA as the abundant component in all the treatments. The concentration of LA was found to increase significantly from 16.6 mg g^−1^ in the initial 6-day-old larvae up to 243 mg g^−1^ in larvae fed with fresh substrates and 365 mg g^−1^ in the case of pre-fermented substrate-fed larvae. Other saturated fatty acids, such as capric acid, myristic acid, palmitic acid, and stearic acid, were present consistently. Among MUFAs, oleic acid was predominant. PUFAs, such as linoleic acid, were found to be present in abundance in all larvae fed with fresh substrates. However, a significant decline was observed in larvae fed with pre-fermented substrates. This may be due to the consumption of UFAs during fermentation. The results of retention analysis revealed a significant accumulation of medium-chain fatty acids, signifying a strong de novo synthesizing ability of BSFL. On the other hand, long-chain UFAs were found to have a retention value of less than 100% that demonstrates the weak ability of BSFL to accumulate long-chain fatty acids (Fig. 3). Some larvae from fresh substrate treatments were found to have α-Linolenic acid (C18:3 n3) in traces, which might be due to the accumulation from the substrates containing α-Linolenic acid like sweet potato and pork (Table S2). Overall, pre-fermentation was found to increase the accumulation of SFAs, particularly LA, thus highlighting the ability of bacteria to convert carbohydrates into required LA precursors like lactic acid, acetyl-CoA, and/or malonyl-CoA. However, from the patterns of key fatty acid accumulation, especially the pre-fermentation treatments with an increase in LA and the depleted long-chain unsaturated fatty acids (UFAs), it is obvious that there are complex metabolic relationships. To further understand the relationships between the fatty acid compositions of the substrates and the larvae, correlation analyses were performed.

**Table 4:** Key fatty acid composition in BSFL biomass in mg/g DM

Fatty acid/sample	IL^#^	C	T1	T2	T3	T4	T5	PC	PT1	PT2	PT3	PT4	PT5
Total crude fat (%)	4.8 ± 0.4	4.7 ± 2.8	21.8 ± 2.1	24.8 ± 2.2	25.2 ± 1.5	27.7 ± 0.9	31.1 ± 0.4	28.2 ± 0.4	33.0 ± 0.6	35.3 ± 0.2	39.5 ± 1.2	41.1 ± 0.1	43.8 ± 0.7
Saturated fatty acids													
Capric acid (C10:0)	0.44 ± 0.03	0.67 ± 0.02^e^	3.9 ± 0.1^d^	8.5 ± 0.3^b^	4.4 ± 0.2^d^	9.4 ± 0.2^a^	0.9 ± 0.1^e^	6.2 ± 0.1^c^	8.9 ± 0.2^ab^	8.4 ± 0.1^b^	9.2 ± 0.3^a^	6.4 ± 0.3^c^	6.6 ± 0.4^c^
Lauric acid (C12:0)	16.6 ± 0.3	22.5 ± 0.9^j^	143.4 ± 2.3^h^	136.4 ± 3.0^i^	138.4 ± 0.7^i^	142.9 ± 2.1^h^	242.6 ± 0.5^f^	220.5 ± 0.7^g^	269.1 ± 0.2^d^	262.7 ± 0.2^e^	323.5 ± 0.4^b^	309.4 ± 0.3^c^	364.8 ± 0.9^a^
Myristic acid (C14:0)	2.6 ± 0.1	4.6 ± 0.1^j^	29.6 ± 0.9^e^	23.2 ± 0.5^h^	26.6 ± 0.3^g^	27.2 ± 0.4^g^	28.2 ± 0.2^f^	16.8 ± 0.2^i^	34.4 ± 0.2^d^	44.7 ± 0.2^b^	37.1 ± 0.2^c^	46.0 ± 0.2^a^	22.8 ± 0.1^h^
Palmitic acid (C16:0)	11.8 ± 0.2	5.7 ± 0.3^l^	15.9 ± 0.3^g^	23.5 ± 0.3^e^	26.4 ± 0.2^d^	30.6 ± 0.2^b^	12.8 ± 0.1^i^	27.7 ± 0.2^c^	6.5 ± 0.3^k^	20.7 ± 0.1^f^	8.1 ± 0.2^j^	14.6 ± 0.2^h^	36.1 ± 0.1^a^
Stearic acid (C18:0)	3.1 ± 0.1	0.6 ± 0.01^f^	1.6 ± 0.1^e^	2.4 ± 0.3^cd^	5.3 ± 0.1^a^	4.3 ± 0.2^b^	2.3 ± 0.2^d^	ND	0.6 ± 0.1^f^	ND	ND	2.8 ± 0.2^c^	ND
MUFAs													
Oleic acid (C18:1 n9)	6.3 ± 0.1	5.8 ± 0.9^i^	12.6 ± 0.1^f^	22.5 ± 0.1^c^	30.6 ± 0.1^b^	32.7 ± 0.2^a^	13.7 ± 0.2^e^	6.4 ± 0.1^i^	6.1 ± 0.1^i^	9.5 ± 0.2^g^	9.5 ± 0.1^g^	17.4 ± 0.2^d^	7.8 ± 0.2^h^
PUFAs													
Linoleic acid (C18:2 n6)	6.3 ± 0.3	6.2 ± 0.4^f^	6.4 ± 0.2^f^	13.3 ± 0.3^c^	15.4 ± 0.3^b^	19.7 ± 0.5^a^	7.4 ± 0.2^e^	4.3 ± 0.3^g^	2.3 ± 0.2^h^	4.4 ± 0.2^g^	3.8 ± 0.2^g^	10.5 ± 0.2^d^	5.8 ± 0.3^f^
∑SFA	34.4 ± 0.1	34.4 ± 0.6^j^	194.8 ± 3.5^i^	196.3 ± 3.3^hi^	201.0 ± 1.2^h^	215.3 ± 1.5^g^	286.8 ± 0.4^e^	271.2 ± 0.4^f^	319.7 ± 0.6^d^	336.8 ± 0.1^c^	379.3 ± 1.3^b^	379.5 ± 0.5^b^	430.2 ± 0.7^a^
∑MUFA	7.3 ± 0.1	6.9 ± 0.7^i^	16.4 ± 0.04^e^	31.6 ± 0.3^c^	35.44 ± 0.04^b^	41.4 ± 0.3^a^	16.4 ± 0.2^e^	6.5 ± 0.1^i^	8.03 ± 0.03^h^	11.4 ± 0.2^f^	12.0 ± 0.5^f^	20.9 ± 0.1^d^	9.2 ± 0.2^g^
∑PUFA	6.3 ± 0.3	6.6 ± 0.4^ef^	6.4 ± 0.2^f^	16.4 ± 0.2^b^	15.4 ± 0.3^c^	20.9 ± 0.8^a^	7.4 ± 0.2^e^	4.3 ± 0.3^g^	2.3 ± 0.2^h^	4.4 ± 0.2^g^	3.8 ± 0.2^g^	10.5 ± 0.2^d^	5.8 ± 0.3^f^
∑UFA	13.5 ± 0.2	13.4 ± 0.9^g^	22.8 ± 0.1^e^	48.1 ± 0.3^c^	50.8 ± 0.3^b^	62.3 ± 0.5^a^	23.7 ± 0.4^e^	10.8 ± 0.4^h^	10.3 ± 0.2^h^	15.7 ± 0.4^f^	15.8 ± 0.3^f^	31.4 ± 0.3^d^	15.0 ± 0.4^f^

The values are expressed in mean ± SD, with decimal places reflecting the precision supported by each measurement’s SD; *p* < 0.05; ND: not detected; ^#^IL: initial 6-day-old larvae used for the treatment; SFA: saturated fatty acids; MUFA: monounsaturated fatty acids; PUFA: polyunsaturated fatty acids; UFA: unsaturated fatty acids

### Fatty acids correlation analysis between larvae and substrate

**Fig. 4:**
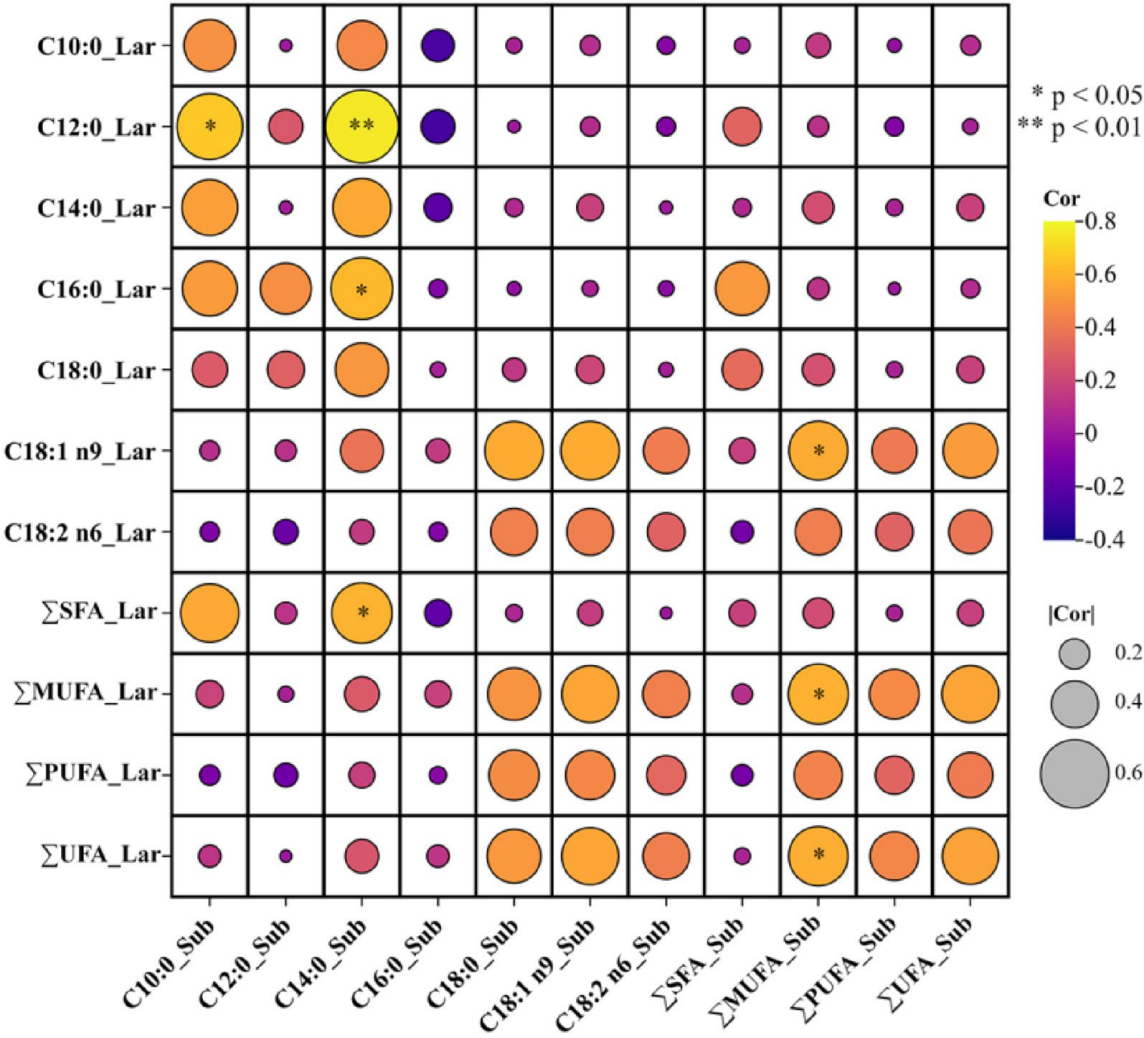
Correlation analysis among fatty acids. (Sub, substrates, and Lar, larvae). *n* = 3; ** *p* < 0.01; * *p* < 0.05

Correlation analysis revealed significant correlations between substrate composition and larval fatty acid profiles (Fig. 4). This strongly suggests that the larval fatty acid is not solely dependent on the substrate fatty acid composition, but rather strongly influenced by metabolic conversions. A strong and statistically significant positive correlation was found for substrate fatty acids and larval saturated fatty acids (SFAs), particularly for C12:0 (LA), C14:0 (myristic acid), and C16:0 (palmitic acid) fatty acids. Notably, larval C12:0 fatty acids showed a statistically significant positive correlation with substrate C10:0 and C14:0 fatty acids. This suggests that both short- and medium-chain fatty acids, as well as their metabolic precursors, contribute to larval LA production. This implies that chain shortening and elongation of fatty acids, where fatty acids can be interconverted through a process called β-oxidation and fatty acid synthase (FAS), are actively occurring. The strong correlation between total substrate SFAs and larval SFAs further supports the selective accumulation and de novo production of saturated fatty acids in BSFL. In contrast, weak or negative correlations were observed between substrate long-chain fatty acids and their larval counterparts. For instance, C18:1 (oleic acid) and C18:2 (linoleic acid) in the substrate had minimal direct association with their larval counterparts, implying that these fatty acids are not accumulated but rather metabolically converted. The decrease in MUFA, such as C18:1, in larvae in spite of their abundance in the substrates implies increased rates of β-oxidation, resulting in the production of acetyl-CoA, which is a precursor in de novo fatty acid synthesis.

**Fig. 5:**
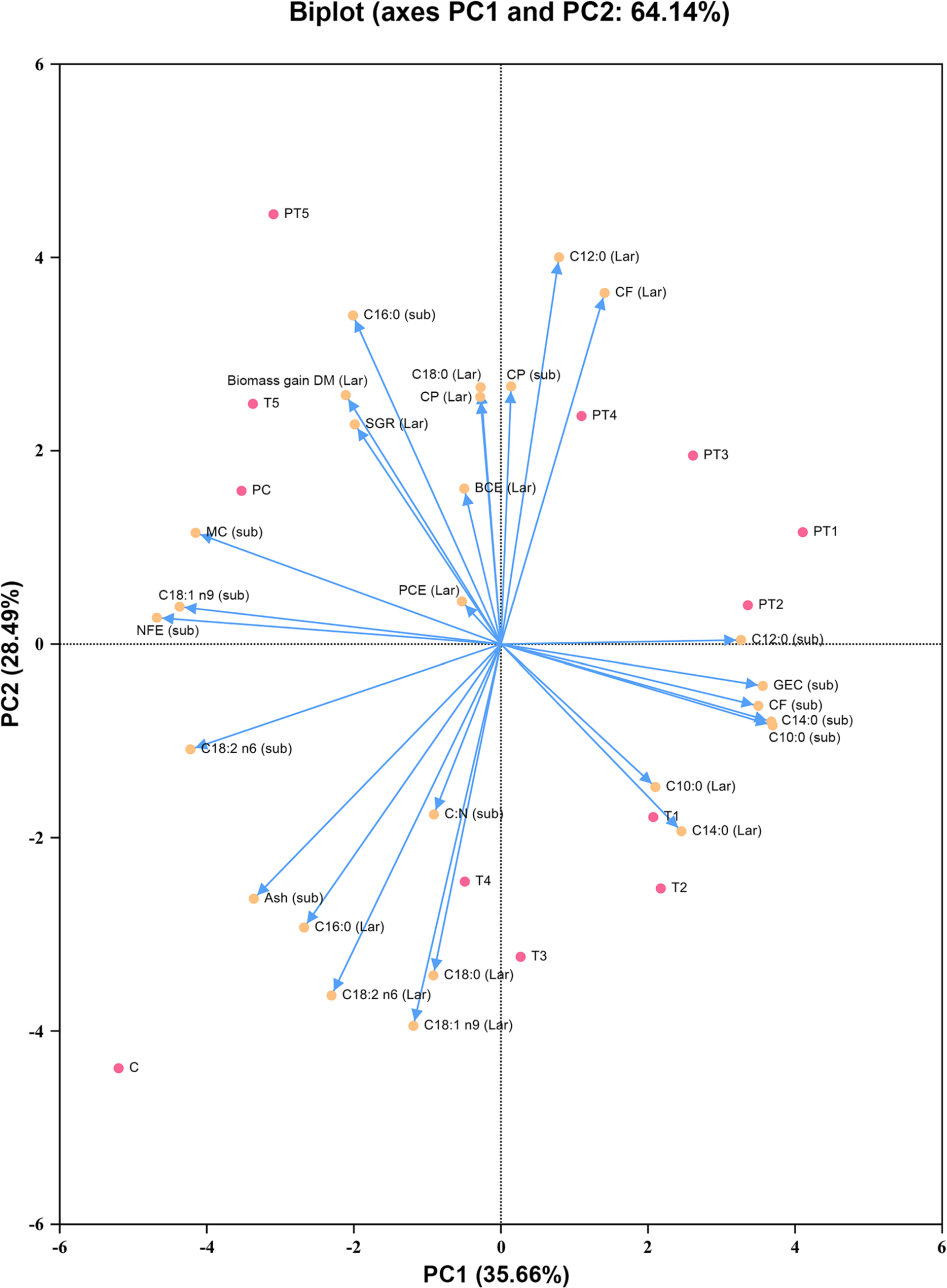
Principal component analysis for different physicochemical parameters of BSFL and the substrates. The interpretation of the data analysis was explained by the Biplot

The PCA biplot has been able to satisfactorily describe the variance (64.14%) in the data set, with PC1 and PC2 contributing 35.66% and 28.49%, respectively (Fig. 5). A clear distinction is seen between pre-fermented treatments and fresh treatments. Principal component analysis (Fig. 5) further supported this evidence, showing that pre-fermented substrates correlated with high larval fat content and LA, while carbohydrate-rich substrates correlated with high larval growth performance.

**Fig. 6:**
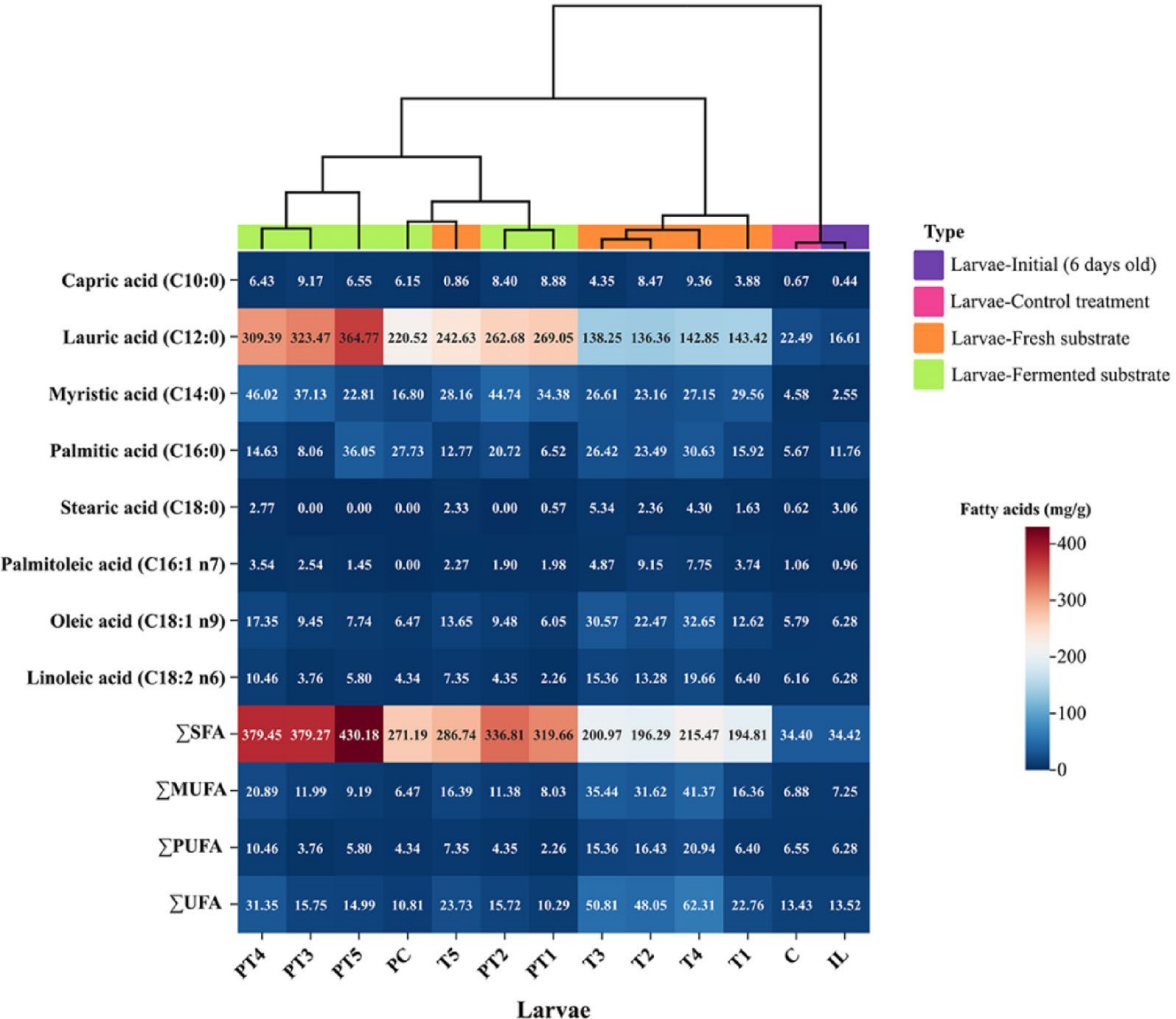
Heatmap for fatty acids variations in larvae and their clustering patterns

The results were further reinforced by the hierarchical clustering analysis (Fig. 6), which distinctly segregated the pre-fermented, fresh, and control samples. The pre-fermented samples were characterized by the presence of elevated levels of SFAs and C12:0, whereas the fresh samples were associated with the presence of elevated levels of UFAs, MUFAs, and PUFAs, such as C18:1 and C18:2. The control and initial larval samples were separated from each other. The results also reveal that the fermentation process induces a metabolic shift in the metabolism of the BSFL, where the levels of UFAs are reduced, and the levels of SFAs are elevated. This is particularly evident in the carbohydrate-rich pre-fermented samples, where the levels of LA are elevated.

The results confirm that the lipid composition of the BSFL is influenced by the substrate and the metabolic shift that occurs during the fermentation of carbohydrates and lipids, where saturated medium-chain fatty acids, particularly C12:0, are elevated in the pre-fermented samples.

## Discussion

### Bacterial consortium and its role in fermentation

Substrate biological treatment using strategic microbial consortia has advanced as a crucial bioprocessing approach for improving BSFL-based bioconversion and organic waste valorization. The selection of the microbes is essentially governed by their hydrolytic enzyme activity, a critical prerequisite for the fermentation of complex organic compounds, especially carbohydrates, proteins, and lipids. In the current study, a consortium of the three isolates (*Bacillus* sp. CS231, *Klebsiella* sp. LSP487, and *Klebsiella* sp. SSP487) was strategically prepared.

The resilience and bioprocess relevance of this consortium design are underscored by gut microbiome evidence: Quan et al. (2023) found the dominance of *Klebsiella* in the BSFL gut as a stable core microbiome across divergent fermented substrates, indicative of a robust host–microbiome alliance that persists irrespective of substrate composition – a critical prerequisite for reproducible bioprocess outcomes. The presence and dominance of *Klebsiella* sp. (Proteobacteria) in BSFL guts at different instar phases have been shown to confer a significant role of the species in improving BSFL growth and performance (Li et al. 2023) without any pathogenic effect, indicative of essential biosafety interest for bioprocessing as an animal feed. Furthermore, in association with insects, *Klebsiella* sp. is a high cellulose-degrading and pectinolytic bacterial genus (Callegari et al. 2020). Therefore, *Klebsiella*, with such a strong gut association for improving the BSFL metabolic pathways, would be a pertinent inoculant for substrate fermentation, especially those that are carbohydrate-rich.

Though capsule formation is an important virulence factor in *Klebsiella*, the low level of mucoidy in the present *Klebsiella* isolates suggests that they are not likely to belong to hypervirulent strains (Struve and Krogfelt 2003). However, rigorous confirmatory studies need to be conducted. Moreover, the strains were only used in a controlled environment, specifically for the purpose of substrate pre-fermentation and not intended for use in the environment or animal feed. The process of fermentation and the bioconversion involving BSFL further reduce the risk of microbes. Future works are intended to confirm the strains using whole-genome sequencing and biosafety considerations before use.

*Bacillus* sp. is a versatile bacterium with various industrial applications, especially related to environmental bioremediation and bioconversion (Qiu et al. 2023; Memon et al. 2025a, b). Given its exceptionally high adaptability and high enzymatic potential to degrade complex organic macromolecules, *Bacillus* sp. would be a worthy choice to support the fermentation process (Xiao et al. 2018; Mazza et al. 2020), rendering it a functional companion in synergy with *Klebsiella* sp.

### Effect of bacterial fermentation on substrate proximate composition and fatty acid profile

The 3-day fermentation allowed for the bacteria to kickstart the bioconversion process and to prevent overutilization of feed resources. The minimal changes in physico-chemical parameters signify the conservation of nutrients and abiotic factors. However, the most prominent changes were seen as selective enrichments of MCFAs (C10:0 to C16:0) *via* utilization of UFAs (especially oleic acid and linoleic acid). This conversion pathway is consistent with Hoc et al. (2020), whereby the UFAs undergo β-oxidation to catabolize into acetyl-CoA and malonyl-CoA, serving as precursors for de novo biosynthesis. The changes in fatty acid composition define the role of bacteria and their enzymes. The restricted supply of oxygen prevents the loss of carbon in the form of CO_2_ and nitrogen in the form of volatilization. Hence, microbial metabolism is the key driving force in the conversion of the substrate without depletion in proximate composition. Bacterial consortia aid in lipase hydrolysis, β-oxidation, and biohydrogenation, which helps convert complex long-chain fatty acids into short-chain fatty acids (SCFAs) and MCFAs. Furthermore, the enrichment of MCFAs in pre-fermented carbohydrate-rich substrates indicates the glycolysis pathway resulting in acetyl-CoA and malonyl-CoA, as anticipated by Liu et al. (2023). In this study, the most notable bioprocess accomplishment is the demonstration that without coconut substrate (the traditional source of LA), the bacterial consortium (*Bacillus* and *Klebsiella*) had either stimulated the larvae’s de novo lipogenesis through stable gut colonization and/or generated bioavailable precursors (MCFA, acetyl-CoA, malonyl-CoA) directly from the sweet potato substrate, paving a dual-path mechanism to LA accumulation with direct implications for sustainable, coconut-independent insect lipid biorefinery.

### Growth performance and nutritional composition of BSFL

In the previous study (Manyapu and Chen 2026), the BSFL gained biomass from carbohydrate-rich guava-based substrate, which is consistent with the present study. The fermentation led to lower substrate reduction, which could be due to acidic pH in the initial periods (Fig. S3). The relatively higher bioconversion in pre-fermented carbohydrate-rich substrates implies that the fermentation of carbohydrate-rich substrates was more balanced and easily consumed by the bacteria than the fat-rich substrates and unfermented substrates, which led to easy nutrient assimilation in the BSFL. The protein conversion was higher in unfermented carbohydrate-rich substrate-fed larvae, and these nutrient-sparing effects were also noted by Rehman et al. (2023). Thus, the BSFL are channeled towards a carbohydrate source when it is abundant and less reliant on protein/fat sources. However, the fermentation induced higher protein conversion in pre-fermented carbohydrate-rich substrates (with the highest sweet potato content), which is in corroboration with Cohn et al. (2022). The increased protein conversion in these treatments implies that the bacterial proteolytic activity partially converted proteins into simple peptides and amino acids, thus increasing the assimilation of nitrogen. The CLR ratio gradually decreased with the decreasing carbohydrate (sweet potato) source, and, interestingly, it was also mirrored in the fresh substrates’ bioconversion. This clearly infers that carbohydrate macromolecules are the primary targets for channeling into acetyl-CoA *via* bacterial hydrolysis, toward lauric acid biosynthesis in the larvae.

The larval NFE (carbohydrate content) shows high accumulation of fermentable sugars and fibers in the BSFL biomass (Manyapu and Chen 2026). Higher NFE is associated with lower fat and protein combination, thus in turn lower GEC and vice versa. The highest GEC observed in pre-fermented carbohydrate-rich substrates (29.2 MJ kg^−1^) versus control larvae (19.2 MJ kg^−1^) demonstrates the superior energy conversion efficiency.

### Substrate bioconversion and its efficiency

The comparable differences in the SRI across all the treatments indicate the voracious feeding nature of the BSFL irrespective of the composition or fermentation: the distinct characteristic of BSFL (Rehman et al. 2023). However, the substantial range in BCE among the treatments (9.8–17.5) shows the influence of the substrate quality and substrate pre-treatment on the BSFL bioconversion ability. The BCE was highest in the pre-fermented carbohydrate-rich treatment because of the availability of readily fermentable sugars for the bacterial treatment, thus enhancing the substrate-to-biomass conversion efficiency (Bothma et al. 2024). The PCE was up to 22.8% in the PT5_Larvae_ associated with enhanced amino acids’ bioavailability and reduced anti-nutritional factors after fermentation (Klüber et al. 2022). The novel metric, the CLR, was the highest in the pre-fermented carbohydrate-rich treatment, which confirms that the bacterial fermentation is a deciding factor for the enhanced LA biosynthesis (Zhu et al. 2019).

### Fatty acid composition of the BSFL

It is obvious to note that LA was the dominating fatty acid in all the treatments, but a huge leap from around 16 mg g^−1^ in initial larvae to 365 mg g^−1^ in pre-fermented carbohydrate-rich substrate-fed larvae was a breakthrough, as a result of unrestricted de novo biosynthesis (Hoc et al. 2020). As a bonus, fermentation converts part of linoleic acid to other forms that can change the metabolic balance and promote SCFA and eventually MCFA synthesis. Similar observations were found by Rossi et al. (2025), where linoleic and oleic acid supported the increased production of LA.

The decrease in the amount of LA in PT4 might indicate a shift in the fatty acid biosynthetic pathway towards chain elongation, leading to the accumulation of more myristic acid. This is also supported by the fact that fatty acid biosynthesis is carried out by adding two-carbon atoms to the fatty acid chain through the action of acetyl-CoA and malonyl-CoA. This might affect the fatty acid chain termination, leading to the formation of either a 12-carbon fatty acid or a 14-carbon fatty acid (Heil et al. 2019). In PT4, it might be able to elongate the LA precursors to form more myristic acid rather than maximizing the accumulation of LA.

The larvae raised in pre-fermented carbohydrate-rich substrate showed lower PUFA content even though the substrates were rich in PUFAs, indicating β-oxidation of UFAs induced by the availability of high acetyl-CoA derived from carbohydrates. This step redirects the metabolic flux to SFA synthesis (Ewald et al. 2020; Liu et al. 2023). The active de novo synthesis can be supported with the key MCFAs retention values that exceed 100%, whereas UFA retention less than 100% show metabolic consumption. The current study involving bacterial pre-fermentation elevates SFAs in BSFL, which contradicts the enrichment of PUFAs in BSFL *via* fungal pre-fermentation (IJdema et al. 2025), demonstrating the significant role of selecting fermentation agents for modifying the BSFL fatty acid profile.

### Fatty acid correlations and metabolic interactions

The significant positive correlation between the substrate and larval MCFAs with weak or negative associated UFAs demonstrates a deeper understanding of BSFL lipid metabolism. The positive association of larval LA with substrate capric and myristic acid suggests active chain elongation and chain length interconversion *via* β-oxidation and fatty acid synthase (Zhu et al. 2019; Liu et al. 2023), thus multiplying the biosynthetic routes for LA biosynthesis.

The positive correlation between substrate CP and larval fat content (in PCA) suggests protein content is essential to maintain metabolic pathways necessary for high-rate lipogenesis (Ewald et al. 2020). The separation between growth parameters (SGR) and fat content (specially C12:0) suggests there is a metabolic trade-off between biomass and fat accumulation, which is regulated by NFE and moisture content in the substrate. Apart from the clear separation of pre-fermented and fresh substrates, the PCA data provide strong evidence supporting the hypothesis that pre-fermentation of the substrate shifts larval metabolism to optimize lipid biosynthesis and saturation. Hierarchical clustering further supports this conclusion from the distinct SFA/UFA-based clusters, which confirms a tractable and scalable metabolic engineering lever for precision lipid biosynthesis in sustainable, insect-based biorefinery platforms.

## Conclusions

Pre-fermentation of carbohydrate-rich substrate *via* the *Bacillus*-*Klebsiella* consortium introduces a novel dimension to this study. Bacterial glycolysis and β-oxidation are the key to the availability of acetyl-CoA and malonyl-CoA, serving as precursors for a remarkable de novo LA biosynthesis of 365 mg g^−1^ (82%). The metabolic transition from unsaturated to saturated medium-chain fatty acids, validated by PCA and hierarchical clustering, corroborates that fermentation enhances carbohydrate-induced lipogenesis. These results are of considerable interest for their industrial applications, as lipids from BSFL containing high amounts of LA are of considerable value in animal feed, antimicrobial agents, biodiesel, and biopolymers.

## Supplementary Information

Below is the link to the electronic supplementary material.

Supplementary Material 1

## Data Availability

Supplementary data are available as Supplementary File 1.

## Abbreviations

BSFL: Black soldier fly larvae
LA: Lauric acid
CLR: Carbohydrate-to-lauric acid conversion ratio
CP: Crude protein
TOC: Total organic carbon
MC: Moisture content
CF: Crude fat
NFE: Nitrogen-free extract
GEC: Gross energy content
SR: Survival rate
SRI: Substrate reduction index
SGR: Specific growth rate
PCE: Protein conversion efficiency
BCE: Bioconversion efficiency
OVD: Overall digestion
PCA: Principal component analysis
UFAs: Unsaturated fatty acids
SFAs: Saturated fatty acids
PUFA: Polyunsaturated fatty acids
MUFA: Monounsaturated fatty acids
MCFAs: Medium-chain fatty acids
SCFAs: Short-chain fatty acids
FAS: Fatty acid synthase
