# Supplementary material for: A strategy for high-yield lauric acid production in Hermetia illucens fed with pre-fermented sweet potato-based substrate
Source: Bioresour Bioprocess. 2026 Jun 12;13(1):90. doi: 10.1186/s40643-026-01085-6 (PMC13263363; doi:10.1186/s40643-026-01085-6)
Supplement: Supplementary file 1 — Supplementary Material 1 [file 40643_2026_1085_MOESM1_ESM.docx]

**Supplementary data**

**A strategy for high-yield lauric acid production in Hermetia illucens fed with pre-fermented sweet potato-based substrate**

Vivek Manyapu ^a,b^ and Yo-Chia Chen ^b,*^

*^a^ Department of Tropical Agriculture and International Cooperation, National Pingtung University of Science and Technology, Pingtung – 912, Taiwan. Email: vivekmanyapu@gmail.com*

*^b^ Department of Biological Science and Technology, National Pingtung University of Science and Technology, Pingtung – 912, Taiwan. Email:* [*ox@mail.npust.edu.tw*](mailto:ox@mail.npust.edu.tw)

*To whom correspondence should be addressed.

Yo-Chia Chen

Phone number: +886-7703202 ext. 5181. Department of Biological Sciences and Technology, College of Agriculture, National Pingtung University of Science and Technology, Pingtung – 912, Taiwan. email: [ox@mail.npust.edu.tw](mailto:ox@mail.npust.edu.tw)

**Table S1. Fatty acid composition of raw substrates in mg/g**

| **Fatty Acid** | **SP** | **DC** | **WB** | **PM** | **PF** | **CF** |
| --- | --- | --- | --- | --- | --- | --- |
| **Total crude fat (%)** | 2.25 ± 0.21 | 33.13 ± 0.41 | 2.74 ± 0.26 | 3.76 ± 0.59 | 73.11 ± 2.05 | 4.7 ± 2.77 |
| **Saturated fatty acids** |  |  |  |  |  |  |
| Caproic acid (C6:0) | ND | 1.53 ± 0.11 | ND | ND | ND | ND |
| Caprylic acid (C8:0) | ND | 29.08 ± 0.17 | ND | ND | ND | ND |
| Nonanoic acid (C9:0) | 0.53 ± 0.04 | ND | ND | ND | ND | ND |
| Capric acid (C10:0) | ND | 23.35 ± 0.22 | ND | ND | 0.22 ± 0.03 | ND |
| Lauric acid (C12:0) | 1.42 ± 0.02 | 160.31 ± 0.65 | 9.89 ± 0.13 | ND | 0.44 ± 0.10 | ND |
| Myristic acid (C14:0) | ND | 61.19 ± 1.17 | ND | 0.67 ± 0.21 | 12.87 ± 0.13 | ND |
| Pentadecanoic acid (C15:0) | ND | ND | ND | ND | 0.15 ± 0.02 | ND |
| Palmitic acid (C16:0) | 6.56 ± 0.15 | 26.45 ± 0.18 | 2.83 ± 0.18 | 10.57 ± 0.16 | 162.26 ± 0.27 | 9.03 ± 0.32 |
| Margaric acid (C17:0) | ND | ND | 0.57 ± 0.03 | ND | 1.68 ± 0.21 | ND |
| Stearic acid (C18:0) | 1.02 ± 0.11 | 10.77 ± 0.18 | ND | ND | 126.18 ± 0.18 | 0.84 ± 0.11 |
| Nonadecanoic acid (C19:0) | ND | ND | ND | ND | 0.73 ± 0.03 | ND |
| Arachidic acid (C20:0) | ND | ND | ND | ND | 2.92 ± 0.09 | ND |
|  |  |  |  |  |  |  |
| **MUFAs** |  |  |  |  |  |  |
| Palmitoleic acid (C16:1 n7) | ND | ND | ND | 1.44 ± 0.19 | 11.44 ± 0.19 | ND |
| Oleic acid (C18:1 n9) | 1.01 ± 0.12 | 16.37 ± 0.16 | 10.44 ± 0.21 | 3.94 ± 0.07 | 253.43 ± 0.55 | 12.63 ± 0.16 |
| Gondoic acid (C20:1 n9) | ND | ND | ND | ND | 7.17 ± 0.28 | ND |
|  |  |  |  |  |  |  |
| **PUFAs** |  |  |  |  |  |  |
| Linoleic acid (C18:2 n6) | 9.44 ± 0.13 | 2.44 ± 0.15 | 3.66 ± 0.21 | 15.83 ± 0.15 | 131.04 ± 0.24 | 24.45 ± 0.28 |
| α-Linolenic acid (C18:3 n3) | 2.55 ± 0.20 | ND | ND | 4.18 ± 0.05 | 9.75 ± 0.23 | ND |
| Eicosadienoic acid (C20:2 n6) | ND | ND | ND | ND | 7.06 ± 0.24 | ND |
| Dihomo-γ-linolenic acid (C20:3 n6) | ND | ND | ND | ND | 0.80 ± 0.02 | ND |
| Arachidonic acid (C20:4 n6) | ND | ND | ND | 0.84 ± 0.10 | 2.92 ± 0.20 | ND |
|  |  |  |  |  | 307.44 ± 0.07 |  |
| ∑SFA | 9.53 ± 0.03 | 312.68 ± 0.22 | 13.29 ± 0.33 | 11.24 ± 0.06 | 272.05 ± 0.10 | 9.87 ± 0.43 |
| ∑MUFA | 1.01 ± 0.12 | 16.37 ± 0.16 | 10.44 ± 0.21 | 5.38 ± 0.12 | 151.58 ± 0.52 | 12.63 ± 0.16 |
| ∑PUFA | 11.99 ± 0.33 | 2.44 ± 0.15 | 3.66 ± 0.21 | 20.85 ± 0.03 | 423.63 ± 0.44 | 24.45 ± 0.28 |
| ∑UFA | 13.00 ± 0.22 | 18.81 ± 0.31 | 14.11 ± 0.02 | 26.23 ± 0.10 | 131.04 ± 0.24 | 37.08 ± 0.43 |

*The values are expressed in mean ± SD; p < 0.05; SP: Sweet potato; DC: desiccated coconut; PM: pork meat; PF: pork fat; CF: corn flour; ND: not detected; SFA: Saturated fatty acids; MUFA: Monounsaturated fatty acids; PUFA: Polyunsaturated fatty acids; UFA: Unsaturated fatty acids.*

**Table S2. Hydrolytic activity of bacterial isolates *via* agar plate assay expressed as enzyme index***

| **Samples/ Enzymes** | **Lipase** | **Protease** | **Amylase** | **Pectinase** | **Cellulase** |
| --- | --- | --- | --- | --- | --- |
| CS181 | 0.00 | 0.00 | 0.00 | 0.00 | 0.00 |
| CS182 | 0.00 | 0.00 | 0.00 | 0.00 | 0.00 |
| CS161 | 0.00 | 0.00 | 0.00 | 0.00 | 0.00 |
| CS162 | 0.00 | 0.00 | 0.00 | 2.58 | 0.00 |
| CS151 | 0.00 | 0.00 | 0.00 | 0.00 | 0.00 |
| CS231 | 0.00 | 1.70 | 1.60 | 2.81 | 2.46 |
| LSP7261 | 3.20 | 0.00 | 0.00 | 0.00 | 0.00 |
| LSP485 | 4.07 | 0.00 | 0.00 | 1.73 | 0.00 |
| LSP487 | 5.38 | 0.00 | 0.00 | 1.67 | 3.77 |
| SSP487 | 6.11 | 0.00 | 0.00 | 1.53 | 3.91 |
| SSP7241 | 3.74 | 0.00 | 0.00 | 0.00 | 0.00 |

**Enzyme index = diameter of the zone of enzyme activity/diameter of the bacterial colony*

**Table S3. Morphological characterization of the selected isolates compared with *Escherichia coli* as a control.**

| **Test** | **CS231** | **LSP487** | **SSP487** | ***E. coli* (Control)** |
| --- | --- | --- | --- | --- |
| **Gram staining** | Positive | Negative | Negative | Negative |
| **Motility test** | Very high | Non–motile | Non–motile | High |
| **Blood test** | α–Hemolysis | γ–Hemolysis, ash grey colony | γ–Hemolysis, ash grey colony | γ–Hemolysis, ash grey colony |
| **Eosin Methylene Blue agar test** | No growth | Pink to purple colony, mucoid, very weak sheen at the initial streaking point. | Pink to purple colony, mucoid, no distinct sheen | Brown to black colony, mucoid, distinct metallic green sheen |
| **Catalase test** | Positive | Positive | Positive | Positive |
| **String test** | Positive, strong | Positive, weak | Positive, weak | Positive, strong |
| **Kovac’s Indole test** | Negative | Negative | Negative | Positive |
|  |  |  |  |  |

**Table S4. Final substrate parameters after 12 days of BSFL treatment**

| **Treatments** | **Moisture content (%)** | **C/N ratio** | **Crude protein (%) ^ns^** | **Crude fat (%)** | **Ash (%)** | **NFE (%)** | **GEC (MJ per kg)** |
| --- | --- | --- | --- | --- | --- | --- | --- |
| **C** | 53.3 ± 2.1^de^ | 11.7 ± 4.3^ab^ | 18.2 ± 2.7 | 4.8 ± 1.4^g^ | 10.1 ± 0.2^a^ | 67.0 ± 4.3^a^ | 17.7 ± 0.5^f^ |
| **T1** | 46.7 ± 1.5^f^ | 17.3 ± 2.4^a^ | 19.9 ± 1.0 | 29.7 ± 2.8^ab^ | 3.7 ± 0.1^e^ | 46.7 ± 3.6^d^ | 24.5 ± 0.7^ab^ |
| **T2** | 51.3 ± 1.2^def^ | 17.1 ± 3.3^a^ | 18.8 ± 2.6 | 20.8 ± 1.8^de^ | 4.5 ± 0.3^d^ | 55.9 ± 4.2^abcd^ | 22.3 ± 0.6^cd^ |
| **T3** | 59.7 ± 2.1^bc^ | 13.9 ± 3.4^ab^ | 19.1 ± 1.2 | 10.7 ± 1.7^f^ | 5.7 ± 0.2^c^ | 64.6 ± 3.1^a^ | 19.8 ± 0.4^e^ |
| **T4** | 57.0 ± 2.0^bcd^ | 13.6 ± 2.1^ab^ | 20.9 ± 1.8 | 7.1 ± 0.5^fg^ | 6.0 ± 0.3^c^ | 66.1 ± 2.0^a^ | 19.0 ± 0.2^ef^ |
| **T5** | 66.0 ± 2.7^a^ | 13.2 ± 0.7^ab^ | 21.2 ± 3.6 | 8.6 ± 3.3^fg^ | 9.1 ± 0.7^b^ | 61.1 ± 6.1^ab^ | 18.9 ± 1.1^ef^ |
| **PC** | 49.3 ± 2.3^ef^ | 9.0 ± 3.9^b^ | 20.4 ± 4.1 | 4.3 ± 0.8^g^ | 10.0 ± 0.3^a^ | 65.3 ± 5.0^a^ | 17.7 ± 0.4^f^ |
| **PT1** | 28.3 ± 3.5^h^ | 15.6 ± 3.8^ab^ | 19.4 ± 3.3 | 33.2 ± 3.0^a^ | 3.4 ± 0.2^e^ | 44.0 ± 2.3^d^ | 25.3 ± 0.6^a^ |
| **PT2** | 33.7 ± 2.1^g^ | 15.1 ± 2.2^ab^ | 20.0 ± 5.7 | 27.5 ± 1.9^bc^ | 3.6 ± 0.2^e^ | 48.9 ± 7.5^bcd^ | 24.0 ± 0.8^ab^ |
| **PT3** | 56.3 ± 3.1^cd^ | 14.2 ± 1.1^ab^ | 19.4 ± 3.0 | 29.2 ± 3.2^ab^ | 3.8 ± 0.1^e^ | 47.6 ± 6.1^cd^ | 24.3 ± 0.9^ab^ |
| **PT4** | 55.7 ± 2.1^cd^ | 15.0 ± 0.8^ab^ | 22.2 ± 4.6 | 23.5 ± 1.9^cd^ | 4.1 ± 0.3^de^ | 50.3 ± 6.2^bcd^ | 23.2 ± 0.8^bc^ |
| **PT5** | 62.7 ± 1.8^ab^ | 10.7 ± 0.9^ab^ | 17.9 ± 1.7 | 17.3 ± 1.2^e^ | 4.7 ± 0.1^d^ | 60.1 ± 0.8^abc^ | 21.4 ± 0.2^d^ |

*The values are expressed in mean ± SD; p < 0.05; ns: not significant at p > 0.05; NFE: nitrogen-free extract; GEC: gross energy content*


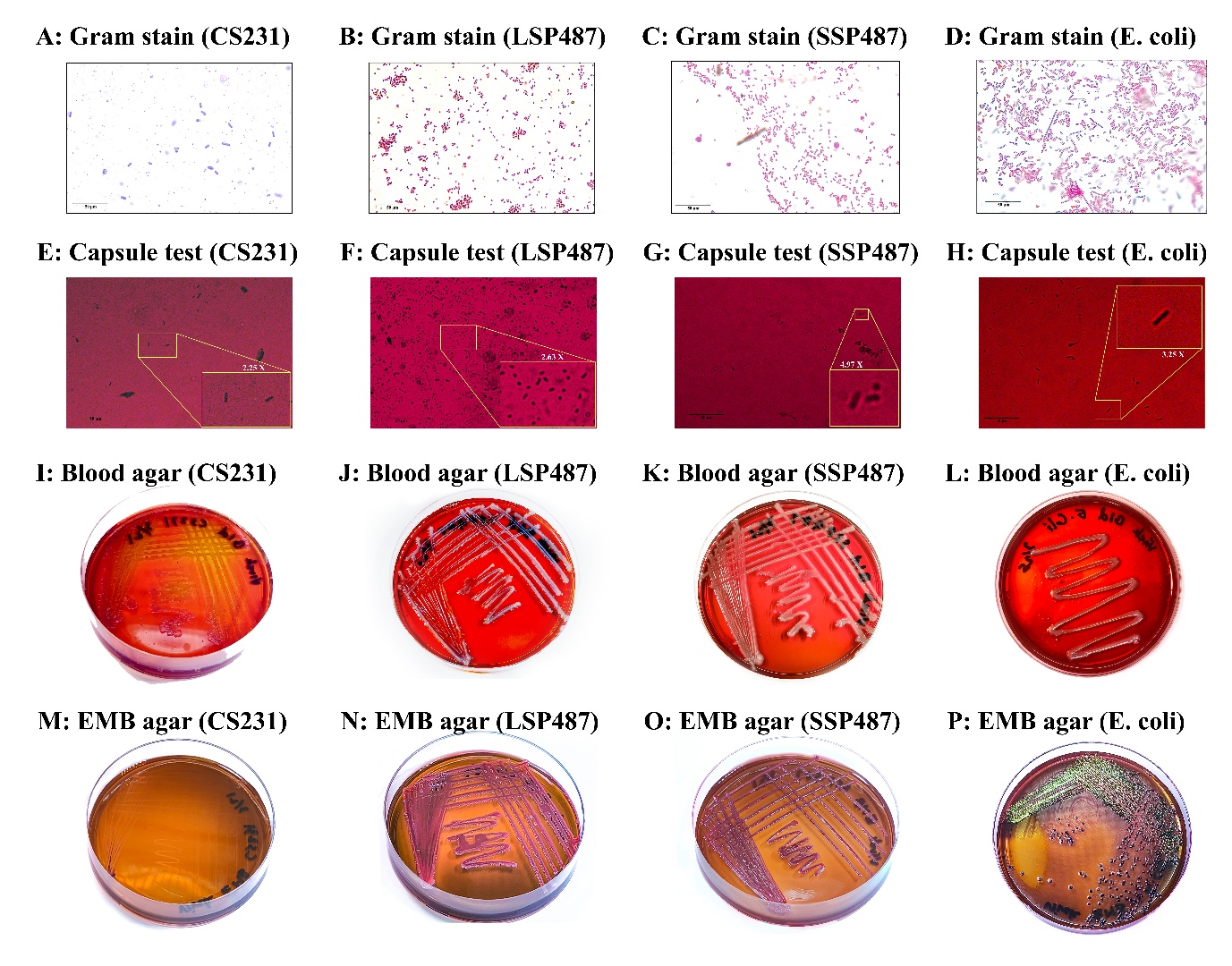
Figure S1. Morphological tests for identification and confirmation of the bacterial isolates. A – D: Gram staining test; E – H: capsule test, capsules observed under TRITC image; I – L: blood agar test for hemolysis; M – P: lactose fermentation test using eosin methylene blue agar


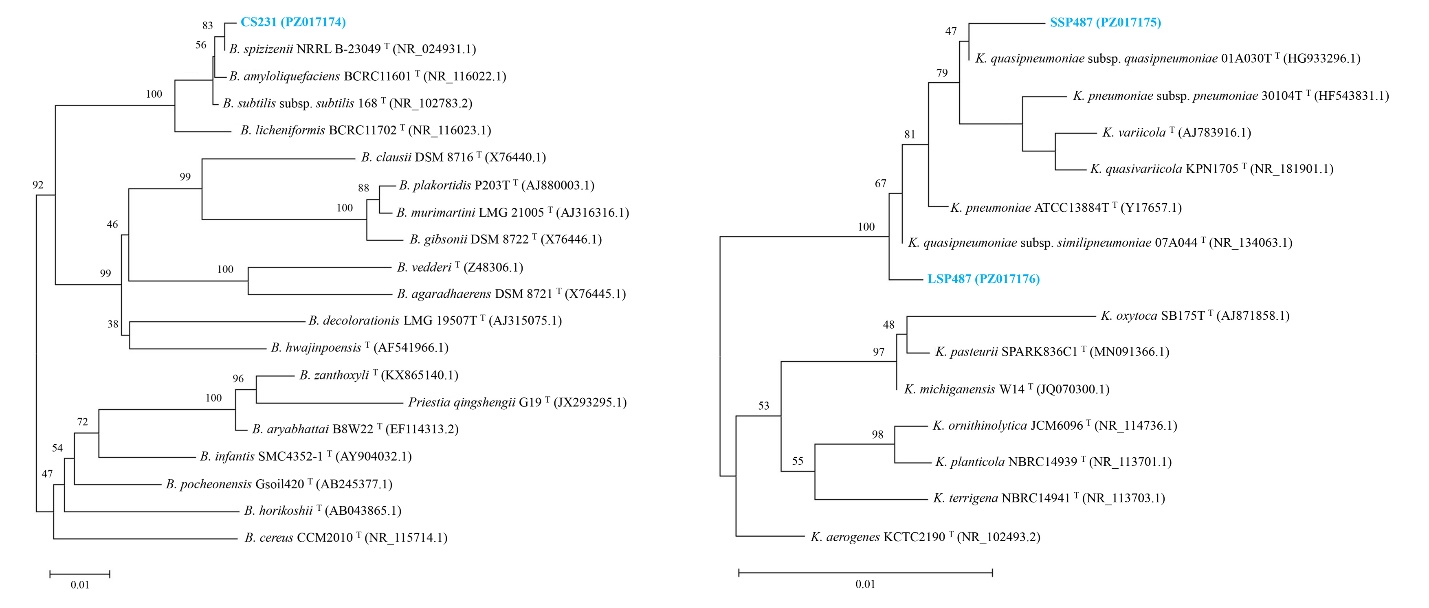


Figure S2. Phylogenetic tree based on 16S rRNA gene sequences showing the relationships among bacterial isolates identified in this study


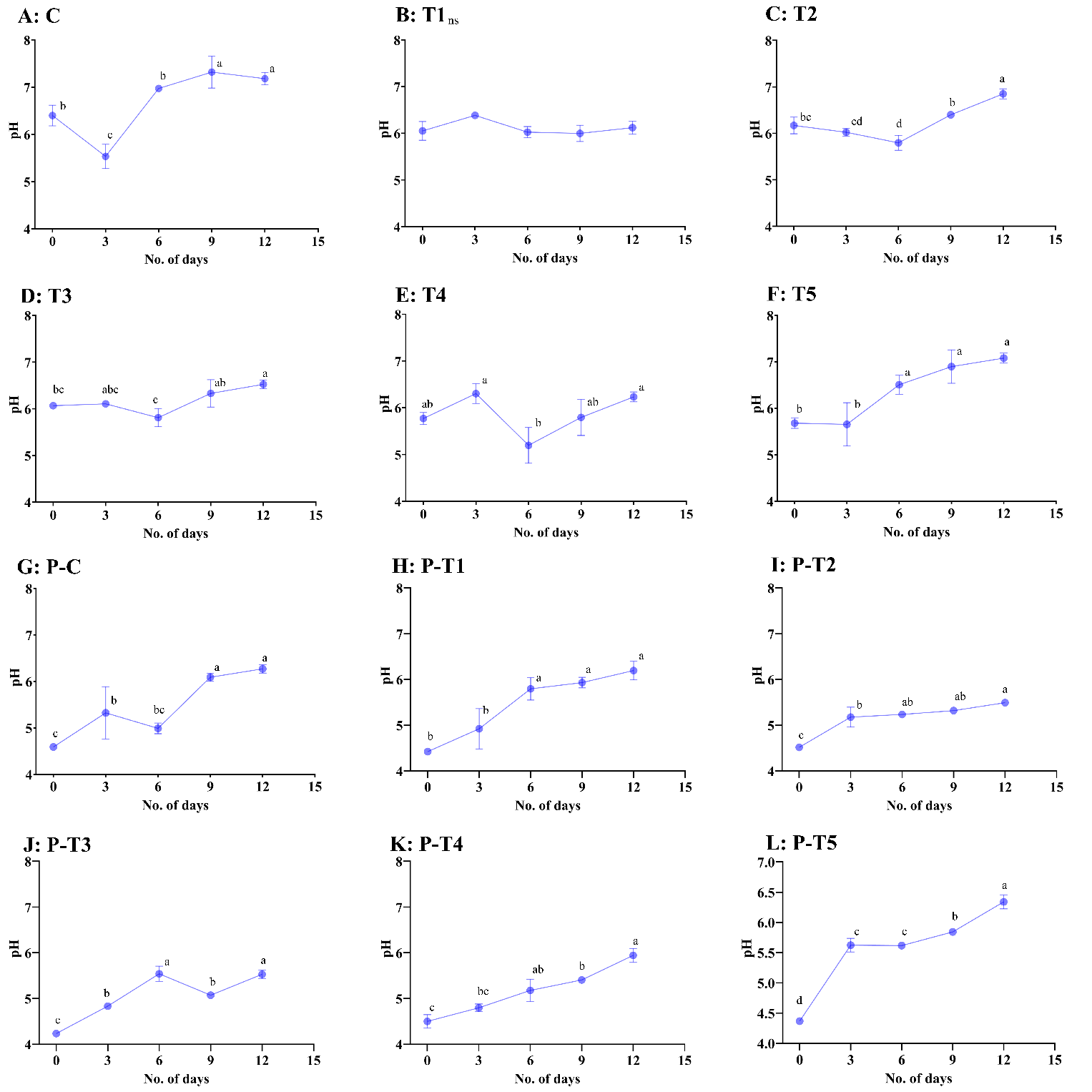


Figure S3. pH variation in the fresh and pre-fermented substrates during the BSFL treatment

*The values are expressed in mean ± SD; p < 0.05*
